# Metabolic Markers Demonstrate the Heterogeneity of Walking Ability in Community-Dwelling Older Adults

**DOI:** 10.1093/geroni/igaf122.788

**Published:** 2025-12-31

**Authors:** Shanshan Yao, Ziling Mao, Megan Marron, Eleanor Simonsick, Venkatesh Murthy, Ravi Shah, Anne Newman

**Affiliations:** University of Michigan, Ann Arbor, Michigan, United States; University of Pittsburgh, Pittsburgh, Pennsylvania, United States; University of Pittsburgh, Pittsburgh, Pennsylvania, United States; National Institute on Aging, Baltimore, Maryland, United States; University of Michigan, Ann Arbor, Michigan, United States; Vanderbilt University Medical Center, Nashville, Tennessee, United States; Vanderbilt University Medical Center, Nashville, Tennessee, United States

## Abstract

Walking ability is important to life quality of older adults. A self-reported walking ability index (WAI) covering the difficulty and ease of walking captures a broader spectrum of walking ability in older persons. Using metabolomics in the Health, Aging and Body Composition study, we identified metabolites related to WAI (0–9, higher scores indicate better walking ability). Among 2,334 participants (mean age 74.6 years, 51% women, 37% Black), 27% scored 0–5, 36% scored 6–8, and 37% scored 9 at baseline (i.e. Year 2). Over 4 years, 52% maintained a stable WAI, 6% improved, while 42% declined (22% 1-2 point and 20% >2 points decline). Using probabilistic index models for cross-sectional and multinomial logistic models for longitudinal analyses, we identified 81 metabolites significantly associated with both poorer concurrent WAI and faster decline, including higher carnitine species, long-chain saturated diglycerides and triglycerides, and TCA cycle intermediates (cis-aconitic, fumaric, malic acids), and lower phospholipids levels. Eighteen additional metabolites were only associated with faster WAI decline: higher short-chain saturated triglycerides and energy metabolism markers (ATP/ADP/AMP), and lower margaric acid and glycine levels. Notably, those with improved WAI, despite poorer baseline WAI and lifestyles, showed more favorable metabolic profiles than others. Metabolites linked to inflammation, energy metabolism, and fatty acid utilization were related to mobility function. Some metabolites might be particularly important for early detection of mobility decline risk. Metabolic profiles may also help identify older individuals (i.e. with improving WAI) with greater metabolic resilience to lifestyle risk factors and health conditions.

